# Screening, Characterization and Evaluation of Mangiferin Polymorphs

**DOI:** 10.1007/s13659-020-00247-z

**Published:** 2020-07-01

**Authors:** Shiying Yang, Qi Zhou, Baoxi Zhang, Li Zhang, Dezhi Yang, Haiguang Yang, Guanhua Du, Yang Lu

**Affiliations:** 1 Beijing Key Laboratory of Polymorphic Drugs, Institute of Materia Medica, Chinese Academy of Medical Sciences and Peking Union Medical College, Beijing, 100050 China; 2 Beijing Key Laboratory of Drug Targets and Screening Research, Institute of Materia Medica, Chinese Academy of Medical Sciences and Peking Union Medical College, Beijing, 100050 China

**Keywords:** Mangiferin, Polymorph screening, Characterize, Stability, Solubility

## Abstract

**Abstract:**

Mangiferin is a compound with many pharmacological activities and exists in many natural products. Anhydrous and hydrate of mangiferin have been reported separately in two literatures, but the polymorphism of this compound has not been realized until this paper. In this study, polymorph screening of mangiferin has been carried out and five forms have been obtained including three new forms never reported. Several solid state characterization methods, such as powder X-ray diffraction, differential scanning calorimetry and thermogravimetry, are used to identify and characterize all of mangiferin forms. The comparison of the crystallographic data and hirshfeld surface analysis were first reported for mangiferin anhydrous and hydrate. Furthermore, the studies on stability, transformation and solubility have been undertaken, the results prompt that form V can be used as the dominant polymorph for the development of innovative pharmaceuticals.

**Graphic Abstract:**

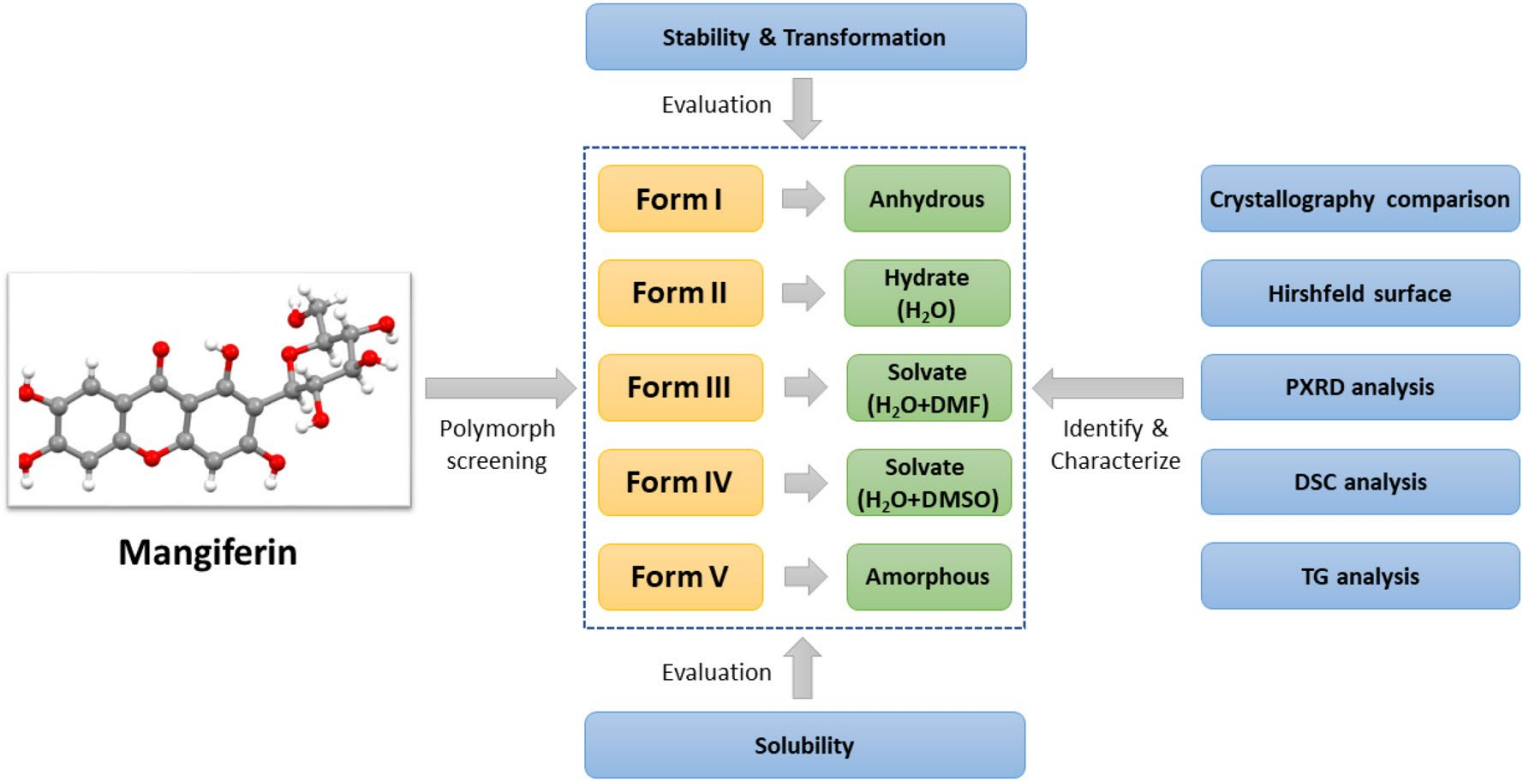

## Introduction

**Fig. 1:**
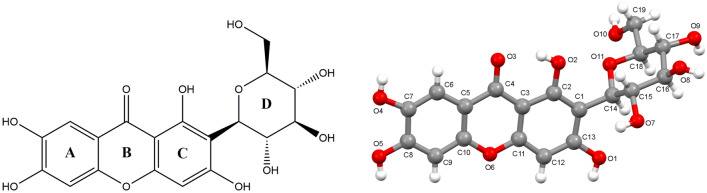
Molecular structure and numbering of mangiferin

Mangiferin (C_21_H_25_NO_4_), also known as alpizarin or chinonin, is a xanthone compound, and its chemical name is 1,3,6,7-tetrahydroxy-2-((3*R*,4*R*,5*S*,6*R*)-3,4,5-trihydroxy-6-(hydroxymethyl)tetrahydro-2H-pyran-2-yl)-9H-xanthen-9-one. Figure 1 showed the molecular structure of mangiferin. The molecular structure of mangiferin includes tetrahydroxydibenzo-γ-pyranone and glucose, forming a c-glycoside. Mangiferin was first discovered as a dye from the roots of mango trees, and it is a secondary metabolite widely existing in natural products. Mangiferin not only exists in the leaves, fruits and barks of mango trees and almonds, but also in other plants, such as *Anemarrhena asphadeloides* Bge, *Belamcanda chinensis* and Gentianaceae of Northeast China. Scientists reported that mangiferin was found in at least 16 other families [1]. Among them, the contents of mangiferin are higher in mango, *Anemarrhena asphodeloides*, *Swertia* and *Pyrrosialingua*. Mangiferin can not only be extracted from natural plants, but also can be obtained by chemical synthesis [2–4] or biosynthesis [5–7].

Pharmacological studies have shown that mangiferin has effects on the central nervous system, respiratory system and cardiovascular system. In addition, it has a wide range of pharmacological activities, such as antioxidant [8, 9], anti-inflammatory [10, 11], anti-virus [12], anti-tumor [13, 14], anti-radiation [15], antibacterial [16], hypoglycemic [17, 18], lowering blood uric acid [19, 20], protecting the liver and choleretic [21], immune regulation [22]. Despite mangiferin has innumerous pharmacological actions and a wide range of plant sources, its poor solubility and bioavailability limit its clinical use. At present, mangiferin is only used as an effective component in several traditional Chinese medicine to treat respiratory diseases such as cough. With the increasing attention of pharmacists on the pharmacological activity and the continuous improvement of the technology for drug development, it is believed that mangiferin will have a good development prospect in the future.

Polymorph screening is an effective technology to improve the solubility and bioavailability of compounds. A compound exists in more than one crystalline form that have different arrangements, different molecular conformations and/or interventions of crystalline solvents or crystalline water, which can be ascribed as polymorphism [23]. Polymorphism is universally exists in solid-state pharmaceutical products and the different polymorphs maybe exhibit different physicochemical properties, such as density, melting point, solubility, stability and bioavailability [24–26]. Therefore, when we are faced with a candidate drug with poor solubility and low bioavailability, polymorph screening is a good choice to improve its pharmaceutical properties. Furthermore, the discovery and patent protection of a new polymorph is of great importance for innovative drugs, from the viewpoints of the development of high-quality and high-efficacy drugs and the protection of valuable intellectual property [27]^.^

Currently, studies on mangiferin reported in the literature are more concentrated in the extraction, synthesis, derivatives, etc., but systematic researches on polymorphism are less concerned. However in fact, mangiferin is a compound with polymorphism. In 2007, Li et al. [28] reported the structure of mangiferin anhydrous form which was named as form I in this paper. In the next year, Cruz Jr. et al. obtained another crystal of mangiferin, which was resolved as a hydrate containing 2.5 molecules crystalline water, named form II [29]. The studies of these two literatures mean that polymorphism indeed exists in mangiferin. Unfortunately, the authors of the latter paper had not retrieved the former one, so there was not any analysis of the difference between the two crystal forms of mangiferin. Since 2008, there is no report on the polymorphs of mangiferin.

In this paper, the systemic crystallization screening for polymorphs of mangiferin has been completed. Five forms of mangiferin have been found and identified, including the anhydrous and hydrate reported before and 3 new polymorphs. All of mangiferin polymorphs were characterized by powder X-ray diffraction (PXRD), differential scanning calorimetry (DSC) and thermogravimetry (TGA). The CIFs were obtained from the the Cambridge Structural Database (CSD), and the comparison of anhydrous and hydrate mangiferin was analyzed. Furthermore, the hirshfeld surface analysis based on these two crystallographic data were carried out successfully. In addition, studies on the transformation among polymorphs and their solubility in vitro had also been completed. These studies are of great significance for the development of mangiferin to an innovative drug.

## Results and Discussion

### Polymorph Screening and Preparation of Polymorphic Samples

Through systematic polymorph screening, five different forms of mangiferin were found, including two forms reported in literatures and three forms found for the first time. During the process of polymorph screening, it was obvious that mangiferin molecules generate the arrangement like form I in nearly 30% experiments, which indicated that form I was the easiest to obtain and maybe the most stable form. Form II was preferentially to generate by recrystallization in aqueous solvent system. Form III and form IV were only obtained in solvent systems containing DMF and DMSO respectively. Form V was amorphous, it could be obtained by heating at high temperatures and mechanical milling. In the process of polymorph screening, mixed polymorphic samples were obtained in 40% of the experiments. After the optimization of preparation parameters, more than 1 g pure polymorphic samples were obtained for each form. The best preparation methods of five polymorphs of mangiferin were listed in Table 1.

**Table 1:** The best preparation methods of five polymorphs of mangiferin

Form	Preparation methods
I	Add 100 mL of 80% methanol solution into 1 g mangiferin to prepare suspension, reflux and stir at 75 °C for 4 h, filter, and dry in vacuum at 60 °C for 2 h to obtain form I
II	Add 200 mL of tetrahydrofuran: water = 1:1 mixed solution into 100 mg mangiferin, heat it at 60 °C for ultrasonic dissolution, filter it, remove the solvent by rotary evaporation at 60 °C, and dry it in vacuum at 30 °C for 2 h to obtain form II
III	Add 2 mL DMF to 200 mg mangiferin, dissolve the sample completely at 60 °C, filter, add 60 mL toluene solution to the filtrate, precipitate in the solution, stand still at 5–10 °C for 24 h, filter, and dry in vacuum at 40 °C for 24 h to obtain form III
IV	Add 2 mL DMSO to 200 mg mangiferin, dissolve the sample completely at 60 °C, filter, add 60 mL *n*-butanol solution to the filtrate, precipitate in the solution, stand still at 5–10 °C for 24 h, filter, and dry it in vacuum at 40 °C for 24 h to obtain form IV
V	Put 5 g mangiferin into the ball mill, set the ball/material ratio to be 6:1, the milling speed to be 400 r min^−1^, grind for more than 9 h to obtain form V

### Single Crystal X-ray Diffraction (SXRD)

The CIF files of mangiferin form I and form II were downloaded from CSD, with CCDC numbers of 819,495 and 649,851 respectively. The crystallographic data and refinement details of both forms of mangiferin are listed in Table 2. From the literature, it was known that mangiferin form I exists in orthorhombic, the chiral space group *P*2_1_2_1_2_1_, and within each asymmetric unit only contains one mangiferin molecule [28]. Form II belongs to the triclinic, the chiral P1 space group with two molecules of mangiferin and five molecules of crystalline water in the asymmetric unit. The two independent molecules in the asymmetric unit have the same configuration but different conformations [29]. However, the comparative analysis of molecular conformation, spatial arrangement and molecular force between the two polymorphs was carried out the first time in this paper. For the convenience of comparative analysis, we had described the mangiferin molecule according to the unified atomic number shown as Fig. 1. In form I, the atoms were directly represented by C or O plus the serial number. In form II, the two molecules in the asymmetric unit were named a and b respectively, so the atoms in a and b were represented by C or O plus the serial number and a or b.

**Table 2:** Crystallographic data for two polymorphs of mangiferin

	Form I	Form II
CCDC no.	819495	649851
Empirical formula	C_19_H_18_O_11_	C_19_H_18_O_11_·2.5H_2_O
Molecular weight (g mol^−1^)	422.34	467.39
Crystal system	Orthorhombic	Triclinic
Space group	*P2*_*1*_*2*_*1*_*2*_*1*_	*P1*
*a* (Å)	7.265 (5)	7.6575 (5)
*b* (Å)	30.086 (4)	11.2094 (8)
*c* (Å)	8.342 (2)	11.8749 (8)
α (°)	90	79.967 (4)
β (°)	90	87.988 (4)
γ (°)	90	72.164 (4)
Volume (Å^3^)	1823.351	955.284
Z	4	2
Density(g cm^−3^)	1.54	1.625
Final *R*, *wR*(*F*^2^) values	0.05, 0.05	0.0471, 0.0471

**Fig. 2:**
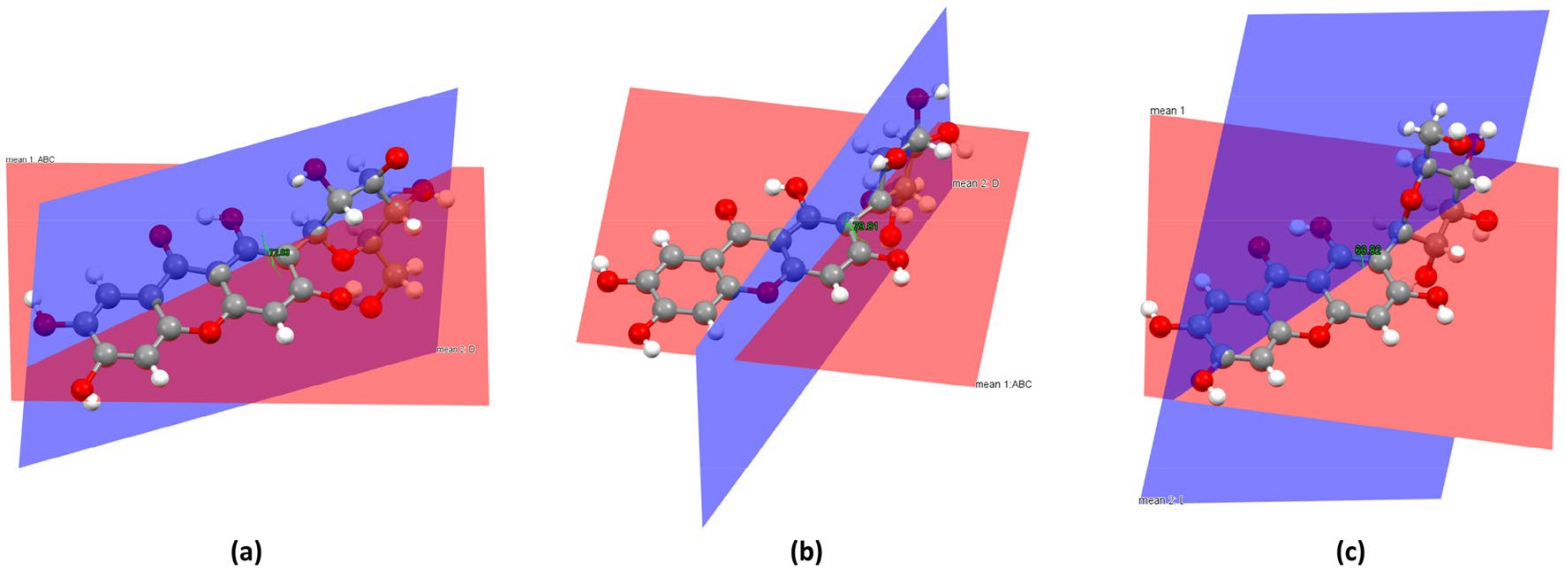
The dihedral angles of two polymorphs of mangiferin, where **a** was for mangiferin molecule in form I, **b**, **c** were for two molecules in form II

The mangiferin molecule was constituted by two parts, which were the xanthone group and the glucopyranosyl group. The xanthone group including rings A, B, and C was rather flat in shape due to conjugation. In form II, the dihedral angles between rings AB and ring C in molecules a and b were 0.97° and 0.71° respectively. But in form I, the dihedral angle between rings AB and ring C was 4.82°. The formation of this conformational difference was related to the intramolecular hydrogen bond in the form I. The intramolecular force formed by O_1_–H⋯O_11_ caused ring C to bend to ring D, with the smallest twist angle value of C_13_–C_1_–C_14_–O_11_, which was 41.81°. The similar intramolecular hydrogen bond did not exist in the molecules of form II, so that rings AB and ring C could maintain good planarity, with the torsion angles of C_13a_–C_1a_–C_14a_–O_11a_ and C_13b_–C_1b_–C_14b_–O_11b_ were 129.33° and − 89.57° respectively. In addition, ring D adopted a chair conformation in both two crystal forms, but the direction between xanthone group and glucopyranosyl group was quite different. The best way to present the differences of glucopyranosyl conformations was to overlap these molecules directly. However, the data of two crystal forms of mangiferin reported in the literature were all relative configurations. According to the CIFs, the relative configuration in the two literatures was just the opposite. Therefore, it was not appropriate to compare the conformational differences between the two crystal forms by overlapping molecules directly, but this did not affect the conformational analysis of two polymorphs. In this paper, the dihedral angels of xanthone group and glucopyranosyl group were used to present their direction differences. The dihedral angle between the plane of rings ABC and ring D in form I was 72.83°, while the dihedral angles were 79.81° and 63.82° in the molecule a and molecule b of form II respectively (Fig. 2).

In both of mangiferin polymorphs, the mangiferin molecules were linked together in a “head-to-tail” mode, but in different detailed manners.

**Fig. 3:**
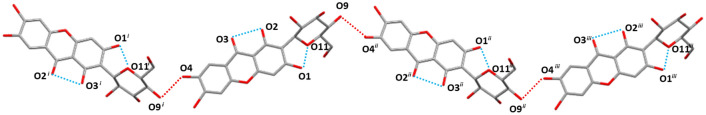
Main hydrogen bonds of O–H⋯O along b-axis in form I (Turquoise dashed lines showed intramolecular actions of O_2_–H⋯O_3_ and O_1_–H⋯O_11_, red dashed lines indicated O_9_–H⋯O_4_ hydrogen bonds. Symmetry codes: *i* = 1/2 − *x*, − 1/2 + *y*, 1 − *z*; *ii* = 1/2 − *x*, 1/2 + *y*, 1 − *z*; *iii* = *x*, 1 + *y*, *z*)

In form I, the head of one mangiferin molecule was linked with the tail of another mangiferin molecule with the symmetry codes (*i* = 1/2 − *x, − *1/2 + *y*, 1 − *z*) by the hydrogen bonds of O_9_–H⋯O_4_, forming a one-dimensional zigzag and infinite chain paralleled b-axis (Fig. 3). The hydrogen interactions of O_4_–O_10_, O_7_–O_10_, and O_7_–O_3_ played an important role in maintaining the stability between chains, forming a layered arrangement perpendicular to the b-axis (Fig. 5).

**Fig. 4:**
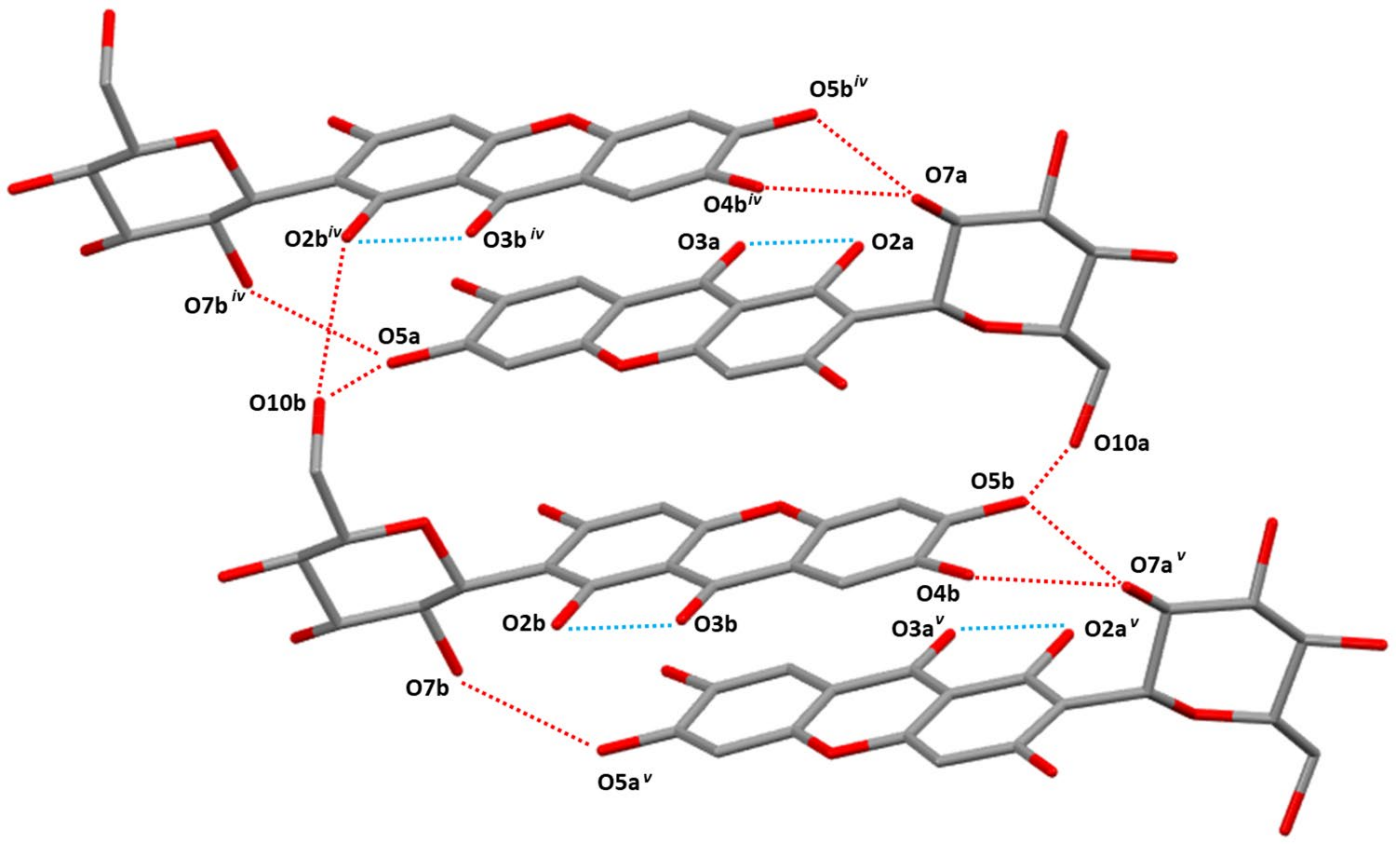
Main hydrogen bonds of O–H⋯O along b-axis in form II (Turquoise dashed lines showed intramolecular hydrogen bonds, red dashed lines indicated intermolecular hydrogen bonds. Symmetry codes: *iv* = *x* − 1, *y*, *z*; *v* = *x* + 1, *y*, *z*)

**Fig. 5:**
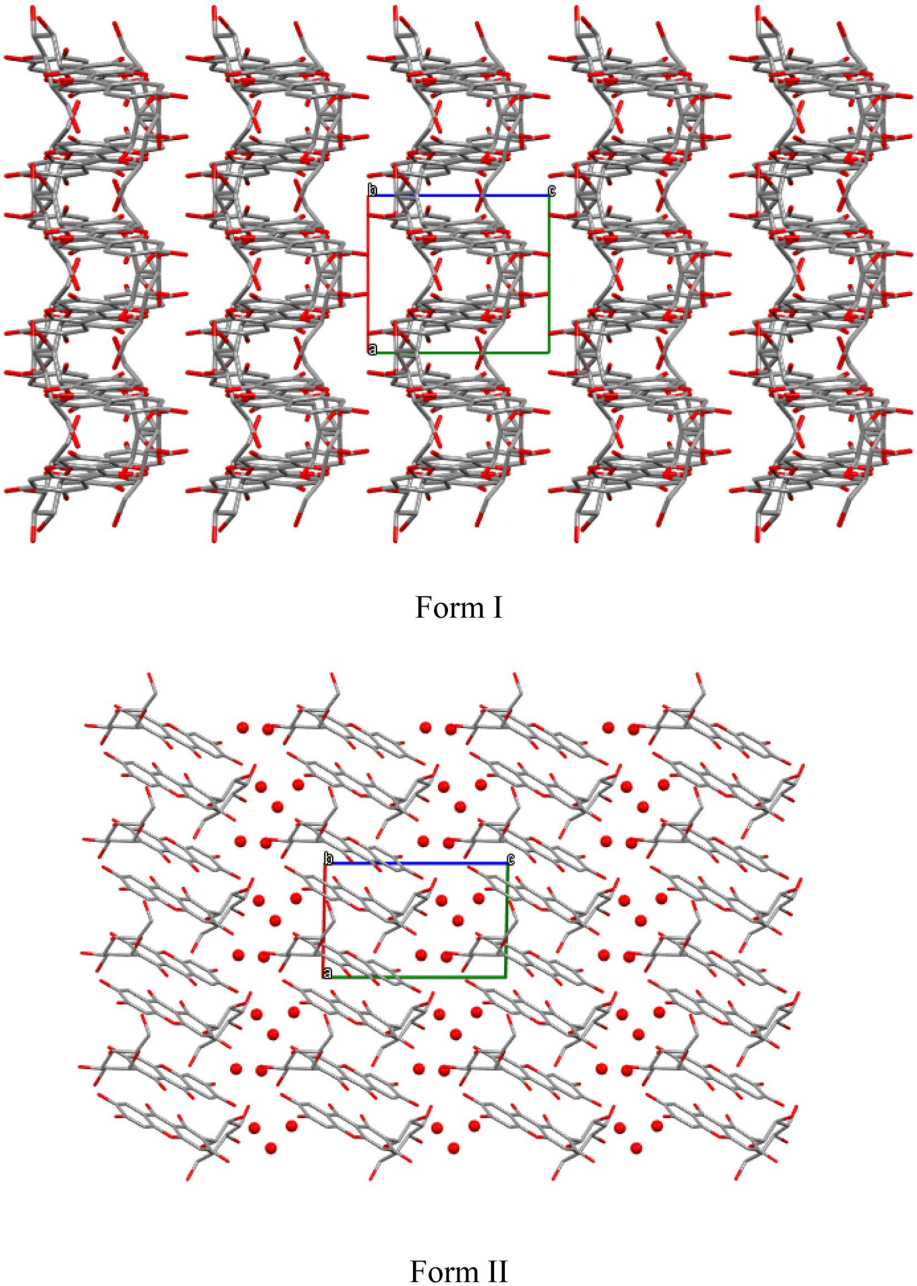
Molecular packing for polymorphs of Mangiferin viewed down crystallographic *b*-axis respectively. (Mangiferin molecules are in stick style, crystalline water molcules are in ball style.)

In form II, two mangiferin molecules in an asymmetric unit were connected in a head-to-tail manner, and the main hydrogen bonds were O_5a_-H⋯O_10b_, O_5b_-H⋯O_10a_. Furthermore, expanded out of the asymmetric unit, the mangiferin molecules were also connected to symmetric molecules by mainly intermolecular H-bonds involving O_7a_–H⋯O_4b_^iv^, O_7a_–H⋯O_5b_^iv^, O_7b_–H⋯O_5a_^iv^, and O_10b_–H⋯O_2b_^iv^. The main hydrogen bonds were shown in Fig. 4. Finally, a layered arrangement along the a-axis direction was formed, and crystalline water molecules were embedded between layers (Fig. 5).

**Fig. 6:**
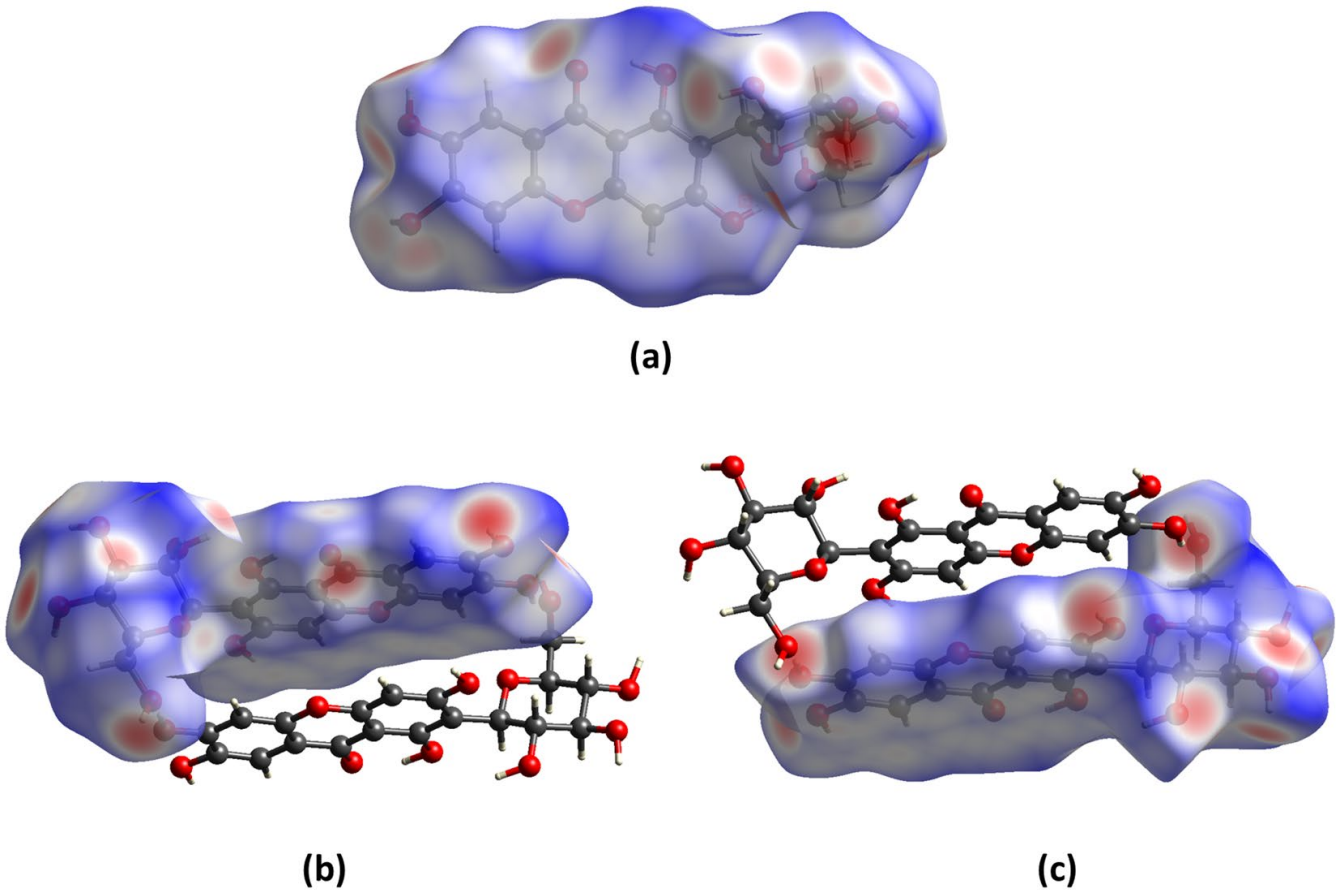
Hirshfeld surface mapped with *d*_norm_ for two polymorphs of mangiferin, where **a** was for mangiferin molecule in form I, **b**, **c** were for two molecules in form II

**Fig. 7:**
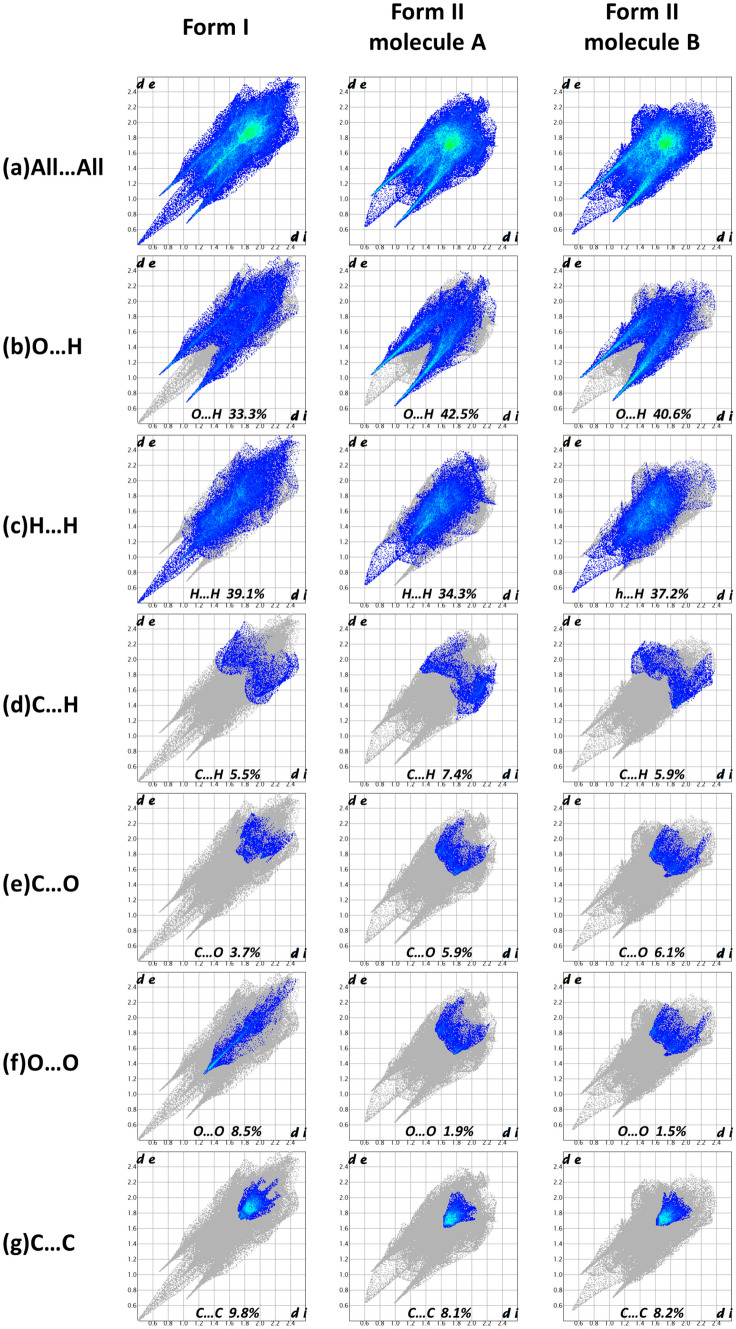
Fingerprint plots of intermolecular interactions of mangiferin form I and form II

Because of many hydroxyl groups contained in the mangiferin molecule, the intermolecular forces were much more complex. So Hirshfeld surface was used to help us analyzing the differences of interactions between two mangiferin forms more clearly. To view molecules as “organic wholes”, Hirshfeld surface is an effective tool to discuss the intermolecular interactions through an unbiased identification of all close contacts, and this approach has been proved highly attractive for the exploration of intermolecular interactions in crystals [30, 31]. The Hirshfeld surface and the 2-dimensional fingerprint plots were unique for a given crystal structure. For forms I and II of mangiferin, the Hirshfeld surface and the 2-dimensional fingerprint plot were calculated using the program Crystal Explore, shown in Figs. 6 and 7. Hirshfeld surface showed the area highlighted with bright red spots, where hydrogen-bonding contacts were formed due to the classical type of hydrogen bonds of O–H⋯O.

From the Hirshfeld surface we can see that almost all oxygen atoms were involved in hydrogen bonding, except for O_6_. In the 2D fingerprint plots, the sharper the spike shape is, the stronger the ability to act as hydrogen bond donor or acceptor is. In both polymorphs of mangiferin, the strongest hydrogen bond donors were O_4_ and O_5_, while the strongest hydrogen bond accepter was O_10_.

**Fig. 8:**
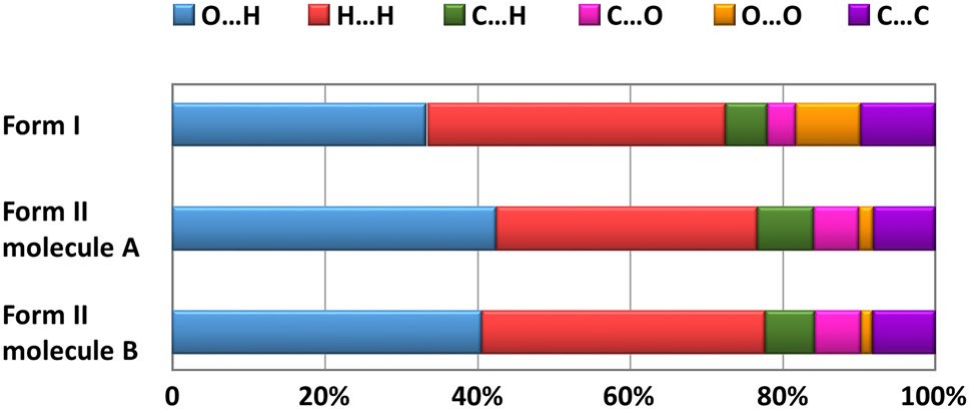
The percentage contributions to the Hirshfeld surface of mangiferin form I and form II

The contribution of different intermolecular interactions to the Hirshfeld surfaces of mangiferin form I and form II were illustrated in Fig. 8. From the percentage contributions to the Hirshfeld surface, we can see that the interactions included many types of contacts, such as O⋯H, H⋯H, C⋯H, C⋯C, C⋯O and O⋯O. In all of these interactions, O⋯H, H⋯H and C⋯C made the main contributions. These interactions played a key role towards the stabilization of the polymorphs in the solid state.

### Powder X-ray Diffraction (PXRD)

**Fig. 9:**
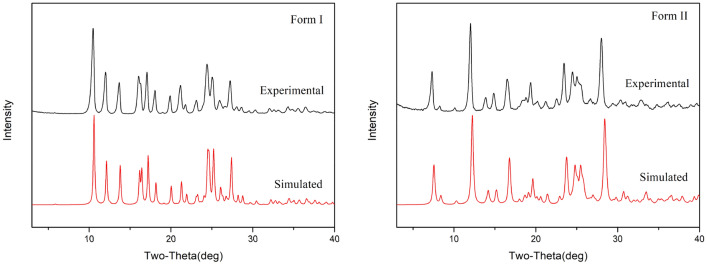
Simulated and experimental PXRD patterns for mangiferin forms I and II

Each crystalline form of a given substance will produce a characteristic PXRD pattern owing to its unique crystal structure. Therefore, simulated powder patterns represent a powerful and fundamental tool to identify new polymorphs. Such patterns can be applied as a standard map to ascertain the pure phases of polymorphs. In this study, the powder X-ray diffraction experiments were proceeded for the five forms of mangiferin. The PXRD patterns of form I and II were compared to their theoretical powder patterns, and the good consistencies indicated that the samples were phases with high polymorphic purity. Simulated and experimental powder patterns of forms I and II are shown in Fig. 9. Besides forms I and II, forms III and IV are in crystalline status, but differ from I and II. Form III was observed the characteristic peaks at 2θ of 6.8°, 8.9°, 20.4° and 25.4°, while form IV showed the peaks at 2θ of 7.5°, 22.7°, 25.2° and 26.4°. These unique peaks proved the formation of new phases, and the absences of characteristic peaks of other forms proved the high polymorphic purity. Form V of mangiferin is amorphous, as it showed a broad peak around 16.4° and 24.4°.

**Fig. 10:**
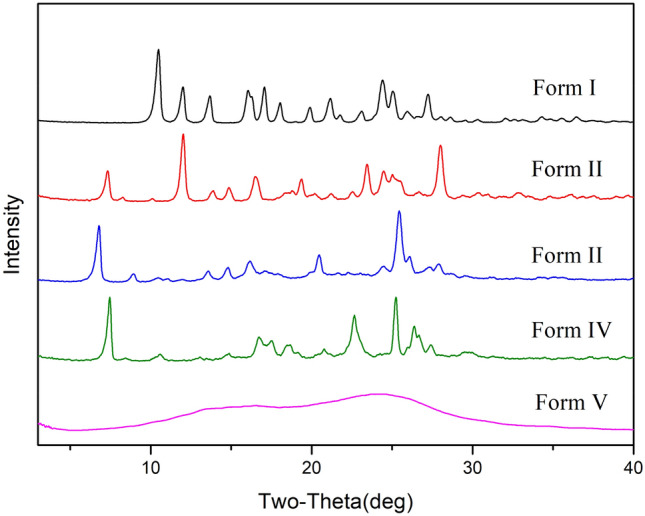
PXRD patterns of mangiferin polymorphs

The five forms of mangiferin have characteristic PXRD patterns, which were well distinguishable from each other, hence in a mixture of crystals of these polymorphs can be easily analyzed. PXRD patterns were showed in Fig. 10.

### Thermal Analysis

**Fig. 11:**
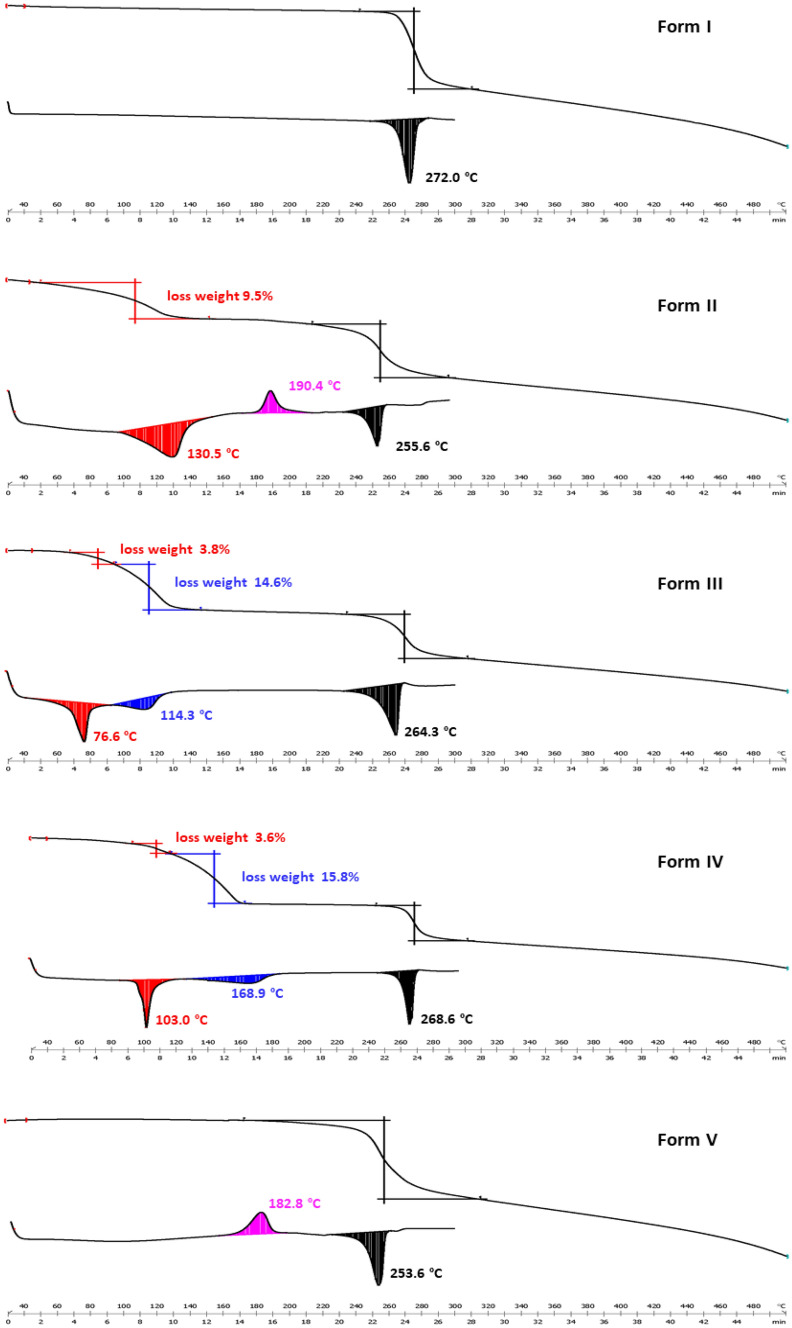
DSC/TGA profiles of mangiferin polymorphs

Differential scanning calorimetry and thermogravimetry analyses were performed to understand the thermal properties of mangiferin forms, and also to identify two things, one is whether the existence of crystal water and solvents in polymorphs of mangiferin or not, the other is how much was there if the crystal water or solvents did exist. The DSC and TGA profiles were shown in Fig. 11. The DSC curve of mangiferin form I showed only one endothermic melting peak which indicated the anhydrous substance. Form V of mangiferin is amorphous, and its DSC also showed the typical characteristics of amorphous, with an exothermic peak at 183 °C and an endothermic melting peak at 254 °C. Form II was a known hydrate and its DSC curve had an endothermic peak at 130 °C which was due to the process of removing crystal water as expected. But it was interesting that an exothermic peak appeared at 190 °C after the process of crystal water removing. That implied us the existing of amorphous transition state during the melting process of mangiferin form II. DSC curves of form III and IV were similar. There were two endothermic peaks at different positions before melting in both forms. This phenomenon suggested that there may be two different kinds of crystallization solvents in the polymorphic sample. In order to further explore the types and amounts of solvents contained in polymorphic samples, thermogravimetric analyses were performed. Mangiferin forms I and V did not have weight loss before their decompositions. Form II had a weight loss of 9.5% from 60 to 150 °C, which was nearly amount to 2.5 molecules of crystalline water. This result was completely consistent with the SXRD analysis of form II. For form III, the TG map also showed similar characteristics to that of the DSC curve during the process of desolvation, and the process of weight loss was phased. 3.8% weight of form III was lost during the temperature range of 50–95 °C, while 14.6% weight lost during 95–140 °C. So we go back to check the recrystallized solvent systems which produced form III. We were delighted to discover the common ground, which was form III only appeared in the preparations methods containing DMF solvent. Therefore, it was speculated that mangiferin form III contained crystalline water and crystalline DMF at the same time. According to the weight loss, 1 molecule of crystalline water and 1molecule of crystalline DMF were calculated to exist in form III. Similar to form III, form IV was only detected in the recrystallized solvent systems containing DMSO, likewise contained 1 molecule water and 1 molecule DMSO by calculations. These judgments were scientific and reasonable no matter in the proportions, boiling points of solvents, temperatures of weight loss and so on.

### Stability Studies and Transformation Among the Six Polymorphs

Mangiferin forms II, III, and IV were proved to be the solvates and hydrates from the thermal analysis. According to their DSC profiles, the transformation experiments were proceeded under different high temperatures. For form II, it is converted to form V under 130 °C for 30 min. But when the temperature reached to higher level, for example 190 °C or 210 °C, form II was converted to form I directly with turning black. The color changes indicated the degradation of the component. In other words, form II undergoes the transient state of form V during the transition to form I. These results of transformation experiments powerfully proved the conjecture about the thermokinetics of form II in the previous section. For form III and IV, they were both transformed to form I when heated at the temperatures higher than their endothermic peaks of the desolvations. Furthermore, form V was also converted to form I at the temperature above 170 °C. Therefore, it can be concluded that form I is the most stable state in thermodynamics.

**Fig. 12:**
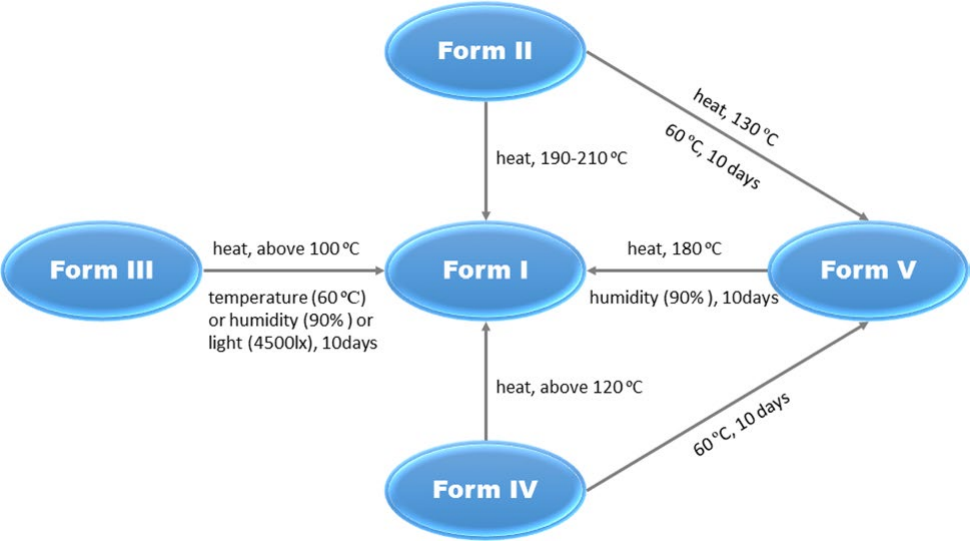
The transformation of mangiferin forms

Besides the transformation experiments, the stabilities of mangiferin five forms under three conditions, temperature (60 ± 1 °C), humidity (90% ± 5%, 25 °C), and light (4500 lx ± 500 lx, 25 °C) were studied according to the Chinese Pharmacopoeia. The results showed that form I was the most stable form and it remained the solid state at above three conditions. Forms II, IV and V were metastable forms, forms II and IV were both converted to V in the condition of 60 °C after 10 days, while form V was converted to I in the condition of 25 °C and relative humidity of 90% ± 5%. Form III was an unstable state and it transformed to form I at three conditions. Figure 12 summarized the transformation of mangiferin forms.

### Solubility Studies

**Fig. 13:**
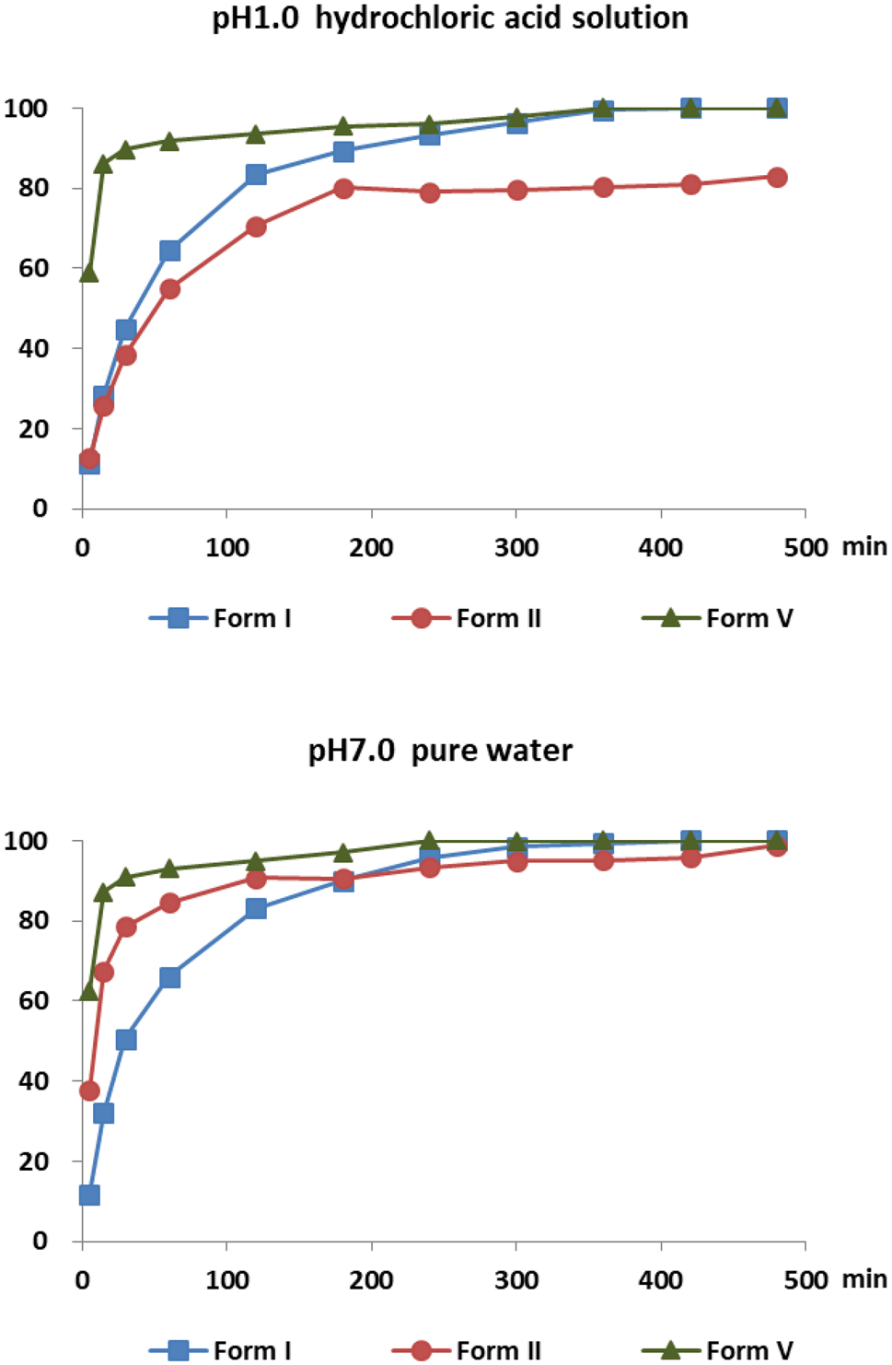
Solubility studies on mangiferin form I, form II and form V

Considering the safety of the polymorph substances, the dissolution tests of forms I, II and V of mangiferin were proceeded in hydrochloric acid solution (pH 1.0) and pure water (pH 7.0). The profiles were shown in Fig. 13. Form V, because of the amorphous state, was dissolved faster than forms I and II in both media at first stage. After 60 min, the amorphous mangiferin reached a platform. In the media of hydrochloric acid solution (pH 1.0), form I had the middle dissolution rate between V and II, and it reached the same solubility as form V, which was 20% higher than form II. In the media of pure water (pH 7.0), form V remained the highest dissolution rate, but form II dissolved a bit faster than form I. Although the differences of their dissolution rates, all of the mangiferin forms almost reached the same solubility after 300 min. Since solubility is one of the most important factors related to drug bioavailability, this study can provide a reference for understanding the bioabsorption properties in vivo of different polymorphs of mangiferin. Further studies about the bioavailability tests of mangiferin polymorphs had been done and published in a Chinese journal, and the results showed form V had the best oral bioavailability as expected.

## Experimental Section

### Materials

Mangiferin raw material was purchased from Shanxi Senfu Biotechnology Co., Ltd. (Shanxi Province, China, batch number: 110410). The chemical purity of this lot is higher than 98.5% mass fractions, which is determined by high-performance liquid chromatography (HPLC). All analytical grade solvents were purchased from the Sigma Aldrich Reagent Company and were used without further purification. High purity water was obtained from a Millipore system with resistivity of 18.2 MΩ cm^−1^.

### Polymorph Screening Experiments

The main aim of polymorph screening was to find the optimal form with the best characteristics for development. In view of the physical and chemical factors, such as solvent system, temperature, speed, humidity, pressure and so on, which may induce the formation of polymorphs, the study tried to obtain as many polymorphs as possible by adjusting the type, quantity and proportion of solvents, temperature and speed during crystallization, humidity, pressure and other factors. The methods of recrystallization, vacuum-rotary evaporation, anti-solvent precipitation, mechanical ball milling, high humidity crystallization and high temperature transformation were used in the polymorph screening of mangiferin. More than 100 screening experiments had been carried out.

### Hirshfeld Surface Analysis

Hirshfeld surface analysis was carried out using the CrystalExplorer program for displaying Hirshfeld surfaces and visualizing intermolecular interactions in molecular crystals. The program accepts a structure input file in the CIF format.

### Powder X-ray Diffraction (PXRD)

Powder X-ray diffraction patterns were recorded on a D/max-2550 (Rigaku, Japan) at room temperature. It used a Copper X-ray source (40 kV and 150 mA) to provide CuK_α_ emission of 1.54184 Å. The divergence and scattering slits were set at 1°, and the receiving slit was set at 0.15 mm. Data was collected from 3° to 80° (2θ) at a step size of 0.02° and scanning speed of 8° min^−1^. Powders for PXRD measurement were obtained by grinding crystalline material in an agate mortar, particle size around 5 μm. Powders were packed into the holder and gently pressed by a glass slide to ensure coplanarity between the sample surface and the surface of the sample holder.

### Thermal Analysis

Differential scanning calorimetry analyses were performed with a Mettler Toledo DSC1 instrument (Mettler, Switzerland). Temperature and enthalpy calibration was performed using an indium and a tin certified reference material. 3–5 mg of each polymorphic sample was heated in the 30–300 °C temperature range at a constant heating rate of 10 °C min^−1^ in aluminum pans with pinhole lids and nitrogen as the purge gas flowing at 50 cm^3^ min^−1^. The crystallize temperatures (*T*_c_, onset), melting points (*T*_m_, onset) were calculated as the mean of three independent measurements determined using STARe software. Thermogravimetric analyses were performed with a Mettler Toledo TGA/DSC1 thermogravimetric analyser (Mettler, Switzerland) to measure the changes in weight of the samples as a function of temperature. The balance was calibrated using standard weights, and the temperature was calibrated by a set of standard materials In, Sn, Bi, Zn, Al before TGA experiment. 5–12 mg of polymorphic samples were heated in Al_2_O_3_ crucibles at a heating rate of 10 °C min^−1^ from 30 to 500 °C under a nitrogen purge of 50 cm^3^ min^−1^.

### Stability Studies

All of mangiferin polymorphs were stored in a drug stability test instrument (SHH-150SD) at three conditions, temperature (60 ± 1 °C), humidity (90% ± 5%, 25 °C), and illumination (4500 lx ± 500 lx, 25 °C), respectively. Periodically, samples were remove from the instrument and subjected to PXRD testing to explore the changes of the systems. These studies were strictly followed the instruction of Chinese Pharmacopoeia.

### Solubility Studies

Solubility studies of all solid-state forms of PPT were performed using the dissolution device RC8MD (Tianjin, China). The dissolution studies were carried out in 900 mL medium at pH 1.0 (0.1 N hydrochloric acid solution) and pure water (pH 7.0), respectively. The rotation speed was set to 100 rpm. For each time point, 2 mL of dissolution medium was collected during the experiments and replaced with 2 mL of fresh pre-warmed medium. The concentration determination performed by high performance liquid chromatography (HPLC) based on a calibration curve improved by a reference method [23] using a HPLC instrument (Agilent 1200, Agilent, USA) at definite intervals (5 min, 15 min, 30 min, 60 min, 120 min, 180 min, 240 min, 300 min, 360 min, 420 min, 480 min) at the reference wavelength (257 nm). Samples were separated by using an Aligent Eclipse XDB C_18_ (150 × 4.6 mm, 5 μm) column. The mobile phase consisted of acetonitrile and 0.4% Phosphoric acid aqueous solution (12:88, v/v), and the flat rate was 1.0 mL min^−1^. The column temperature was set to 30 °C and the injection volume was 5 μL. Validation of the HPLC developed method was done for seven parameters including Linearity, Range, Accuracy, Precision, Limit of Detection (LOD), Limit of Quantitation (LOQ) and Robustness. The Linearity was analyzed by six standard solutions (50, 100, 200, 250, 350, 500 µg mL^−1^), and the regression equation found was *Y* = 14.732*X* + 40.387, *R*^2^ was 0.9994, and the range was from 50 to 500 µg mL^−1^. The Relative standard deviation (RSD) of instrument precision was 0.10%, and the RSD of method precision was 0.30%. LOQ was selected to be the concentration that gave S/N ratio between 10 and 20, and LOD was selected as concentration that gave S/N ration between 3 and 10. The LOQ and LOD were 0.13 µg mL^−1^ and 0.49 µg mL^−1^, respectively. Robustness was investigated by testing the influence of small changes in HPLC conditions as change in flow rate and change in mobile phase composition, and the results indicates that the method completely met the requirements of quantitative analysis.

## Conclusion

In the current study, the investigation of polymorphism of mangiferin was successfully undertaken. Screening all possible polymorphs and measuring their relative stabilities had become crucial steps during drug and food supplements development [32]. Five polymorphs of mangiferin had been screened and prepared, including two reported forms and three new forms. The comparison of the crystallographic data of mangiferin form I and form II was analyzed for the first time. Hirshfeld surface was also used to clarify the intermolecular interactions. Furthermore, PXRD, DSC and TGA methods were used to identify and characterize five forms of mangiferin, and the thermal analysis gave out the important evidences for the determination of the type of solvates (form III and form IV). In addition, the studies on polymorph stability and transformation were carried out, and form I was proved to be the most stable form while form V acted as a metastable form. According to the IDR studies, we can see that amorphous had the fastest dissolution rate, which means that the polymorphs should be considered to beneficial for the pharmaceutical quality and efficacy. Considering all of the evaluation results, form V can be used as the dominant polymorph for subsequent development of innovative pharmaceuticals.

In general, this paper will provide valuable research data of solid states for the drug development with mangiferin as the active pharmaceutical ingredient.
